# Surgery of Schwannoma in the upper limb – sensitivity and specificity of preoperative magnetic resonance imaging and relation between tumour size and symptoms

**DOI:** 10.1186/s12891-023-06838-4

**Published:** 2023-09-07

**Authors:** Emanuel Istefan, Johannes Belstock, Lars B. Dahlin, Erika Nyman

**Affiliations:** 1 Department of Biomedical and Clinical Sciences, Linköping University, Linköping, 581 85 Sweden; 2 Department of Translational Medicine – Hand Surgery, Lund University, Malmö, 205 02 Sweden; 3 Department of Hand Surgery, Skåne University Hospital, Malmö, 205 02 Sweden; 4 Department of Hand Surgery, Plastic Surgery and Burns, Linköping University Hospital, Linköping, 581 85 Sweden

**Keywords:** Peripheral nervous system, Neoplasms, Peripheral nerve, Schwannoma, Upper limb, Surgery, Magnetic resonance imaging

## Abstract

**Background:**

Benign peripheral nerve tumours consist of different types, most commonly Schwannomas. Preoperative Magnetic Resonance Imaging (MRI) is commonly performed before surgery and Pathoanatomical Diagnosis (PAD) confirms the diagnosis. Our aims were to study the utility of MRI and the relation between tumour size and symptoms.

**Methods:**

Retrospectively, patients, surgically treated for benign nerve tumours between 2008 and 2019, were identified and preoperative MRI, with measurement of tumour size, PAD, symptoms, peroperative details, and symptomatic outcomes of surgery, were analysed.

**Results:**

The sensitivity and specificity to correctly identify Schwannomas with preoperative MRI were 85% and 50%, respectively, based on 30 Schwannomas and nine neurofibromas that were identified. Tumour size did not affect the presence of preoperative symptoms, but patients with sensory dysfunction at last follow-up had larger Schwannomas (p < 0.05). Symptoms as a palpable tumour, paraesthesia and pain improved by surgical excision (p < 0.001, p < 0.001 and p < 0.012, respectively), but sensory and motor dysfunction were common postoperatively. No malignant peripheral nerve sheath tumours (MPNST) were found. Using a surgical microscope, instead of only loop magnification, lowered the risk of perioperative nerve injuries (p < 0.05), but did not further diminish postoperative symptoms.

**Conclusions:**

Early and accurate diagnosis of Schwannomas is valuable for adequate presurgical preparation and prompt surgical intervention. Preoperative examination with MRI has a high sensitivity, but low specificity; although recent advancement in MRI technology indicates improvement in diagnostic precision. Surgical excision is preferably performed early in conjunction with symptomatic debut to improve outcome.

## Background

A Schwannoma is a benign nerve sheath tumour and the most common tumour in the peripheral nervous system [1–5]. Because Schwannomas originate from Schwann cells, they are found to grow around nerve fibres, extending from a fascicle, and forming a tumour capsule [6]. A Schwannoma presents in over 90% of cases as a solitary tumour and can develop in any anatomical region [7–9]. Of solitary Schwannomas, 19% affect the upper limb and frequently the volar regions of the extremity due to higher density of nerves [1, 7, 10]. Non-solitary Schwannomas are often associated with multiple neoplasia syndromes, such as neurofibromatosis type 2 and Schwannomatosis [11–13]. Depending on the tumour expansion affecting the nerve fibres, patients with Schwannoma may experience paraesthesia and pain as well as impaired sensory and motor function [1, 3, 10, 14–18]. The treatment is surgical removal of the tumour mass without injuring healthy surrounding nerve tissue, but excision may still harm the nerve fibres and lead to augmentation or development of new symptoms [17, 19–21]. For this reason, microsurgical technique under high magnification has been recommended to minimise this risk [1, 5, 8, 15, 22–24]. Early surgical treatment has been related to shorter postoperative recovery [8].

Magnetic Resonance Imaging (MRI) can preoperatively characterise a Schwannoma, its size, origin, and relationship to adjacent structures in addition to demonstrate tumour engagement of the nerve [22, 25]. Recent development in MRI-technology may also provide the treating surgeon with additional information regarding tissue structure [26–28]. However, there may be diagnostic difficulties in the differentiation between Schwannoma and other nerve sheath tumours, such as neurofibroma, atypical neurofibroma and lipofibromatous hamartoma as well as malignant peripheral nerve sheath tumours (MPNST), using the conventional MRI technique [10, 25, 29, 30]. Performing an MRI entails an economic expense and might extend the time to surgical removal of the tumour mass. Despite this, MRI is the clinical gold standard for preoperative diagnosis and planning of surgery at many units and is favourable over ultrasonography regarding the preoperative diagnosis [1, 10]. Perioperatively, the tumour is sent for Pathoanatomical Diagnosis (PAD) for verification.

The present aims were to evaluate utility of MRI for diagnosis of Schwannoma in the upper limb and tumour size to pre- and postoperative symptoms.

## Methods

Patients, surgically treated between January 1st 2008 and December 31st 2019 for benign nerve tumours in the upper limb at the department of Hand Surgery, Plastic Surgery, and Burns, Linköping University Hospital, Linköping, Sweden, were identified by screening medical records with the ICD-10 code D36.1 (benign neoplasm of peripheral nerves and autonomic nervous system). Data were collected by a medical student and an orthopaedic surgery resident (EI and JB), not involved in the treatment of any of the patients, and analysed retrospectively. Preoperative MRIs were blinded, and all PAD-verified Schwannomas were measured regarding length and width of the tumours.

Data as age, sex, smoking, engaged side, anatomical location of tumour and specific nerve engagement were collected. Furthermore, preoperative symptoms, duration of symptoms until surgery, postoperative symptoms as well as perioperative data, including use of microscope, if enucleation was possible, any notes of perioperative nerve injury or unexpected complications during or after surgery, such as major bleeding or infection, were also noted. As for PAD, additional variables were gathered, such as interpreted type of tumour and radicality.

Normally distributed data, such as age, are presented as mean and standard deviation (SD), whilst not normally distributed data are presented with the median and interquartile range [IQR] as the 25th − 75th percentiles. Statistical analyses to discover differences between groups were performed using independent-samples T test and Mann-Whitney U test. For categorical comparisons between presence of symptoms preoperatively and at last follow-up visit, symptoms regarding microsurgical technique and microsurgical technique and perioperative nerve injury, the dependent McNemar’s test was utilised. Correlation between tumour length and width was assessed with Spearman’s correlation. Diagnostic sensitivity, specificity, positive predictive value (PPV) and negative predictive value (NPV) were estimated for MRI identifying Schwannoma compared to PAD. The sensitivity was estimated as the ratio between PAD-verified Schwannomas, where preoperative MRI indicated Schwannoma, divided by all PAD-verified Schwannomas. The specificity was defined as the number of cases, where the PAD confirmed the preoperative MRI not suspecting Schwannoma, divided by all the cases, where PAD rule out a Schwannoma. PPV was estimated as the cases, where the preoperative MRI correctly identified a Schwannoma, divided by all the cases, where MRI suspected a Schwannoma. Likewise, NPV was calculated as the cases, where the preoperatively MRI correctly rejected Schwannoma, divided by all the cases, where MRI rejected a Schwannoma. Data management and statistical analyses were made with the statistical software SPSS (IBM Corp. Released 2017. IBM SPSS Statistics for Macintosh, Version 25.0. Armonk, NY: IBM Corp.).

## Results

### Patient characteristics

**Fig. 1:**
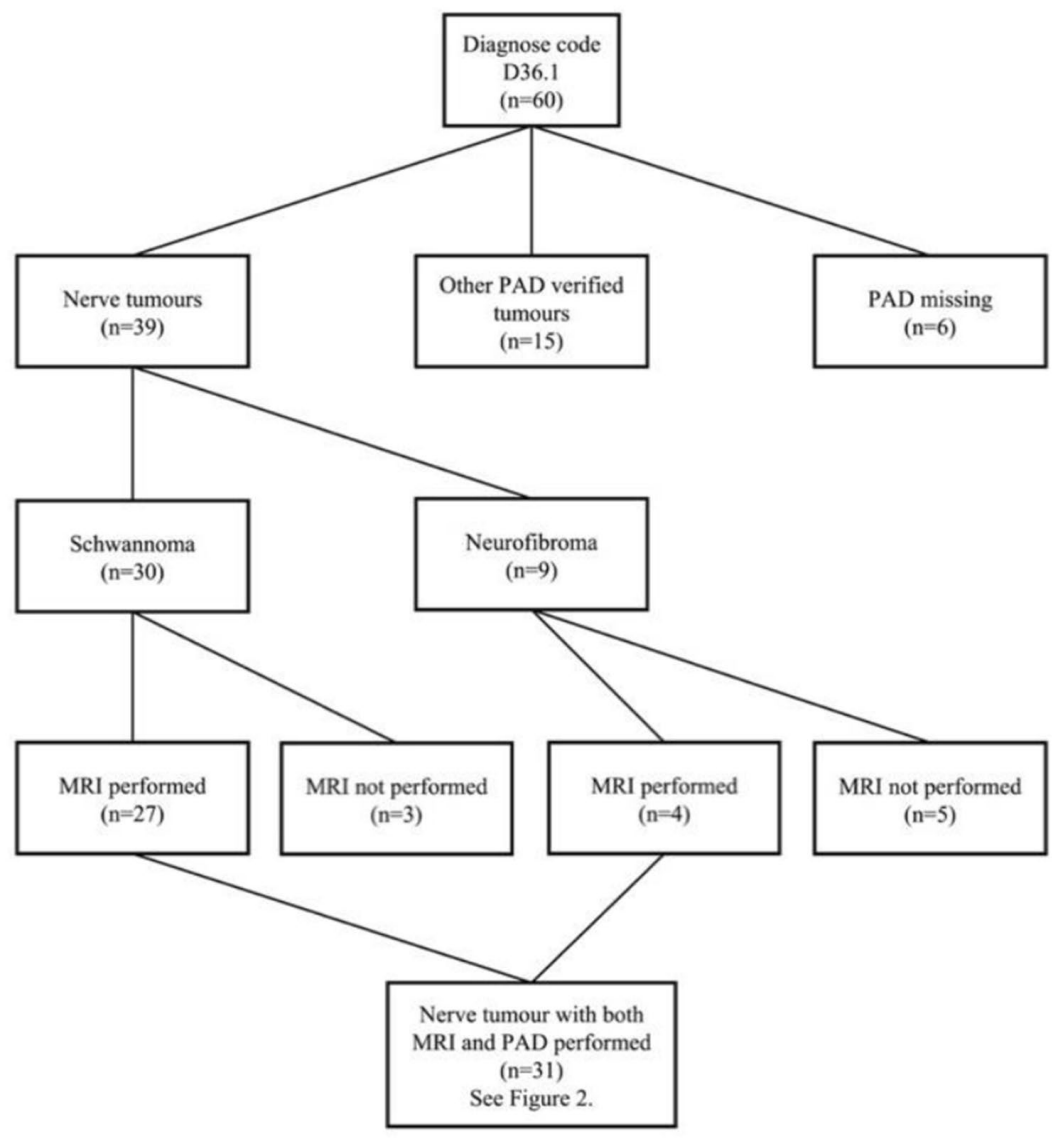
Flowchart of patients having surgery for a suspected benign nerve tumour in the upper limb based on ICD-10 code D36.1. PAD = Pathoanatomical diagnosis; MRI = Magnetic Resonance Imaging

In total, 60 patients, surgically treated for a suspected benign nerve tumour in the upper limb, were identified. Among these 60 included patients, 30 had Schwannoma, 9 had neurofibroma, 15 had another PAD-verified diagnosis, such as glomus tumour (two patients), malignant spindle cell tumour, tendon sheath fibroma, pyogenic granuloma, fibroma, palmar fibromatosis (Dupuytren´s contracture; three patients), angioleiomyoma, benign vessel tumour, lymphangioma, giant cell tumour, vessel anomaly and inconclusive PAD, while in six patients no PAD was performed. Schwannomas and neurofibromas constituted all PAD-verified nerve tumours, as no perineuriomas nor lipofibromatous hamartomas, or other nerve tumours, were present. An overview of the included patients is provided in Fig. 1. No malignant peripheral nerve sheath tumours (MPNST) were found or included.

In the whole population (n = 60), there was an equal sex distribution (male:female 31:29), a mean age at surgery of 46 years, ranging from 13 to 93 years [SD 17 with no significant differences in age between sexes (p = 0.50)]. Among the 30 patients with a PAD-verified Schwannoma, the mean age was 48, ranging from 19 to 79 years [SD 18], at surgery. Sub-analysis of patients with a Schwannoma showed that a slight majority were males (n = 18, 60%) with no significant differences in age between sexes (p = 0.88) (Table 1).

**Table 1:** Characteristics of patients surgically treated for suspected nerve tumours in the upper limb

	All patients ^a^ (n = 60)	Schwannoma (n = 30)	Neurofibroma (n = 9)	Others ^b^ (n = 15)
Sex (Male:Female)	31 (52):29 (48)	18 (60):12 (40)	3 (33):6 (67)	7 (47):8 (53)
Age (years)	46 (17)	48 (17)	45 (22)	45 (16)
Symptom duration until surgery (months) ^c^	18 (26)	12 (30)	18 (17)	24 (24)
Smoking (Yes:No) ^d^	5 (9):52 (91)	2 (7):27 (93)	1 (12):8 (88)	1 (8):13 (92)
Location of tumour ^e^				
Hand	25	7	3	14
Forearm	15	12	1	1
Elbow	4	2	1	0
Upper arm	9	6	2	0
Shoulder	4	2	2	0
Brachial plexus	1	1	0	0
Affected nerve ^f^				
Median nerve	17	15	0	1
Ulnar nerve	14	10	2	1
Radial nerve	1	0	1	0
Other	15	3	2	10
Engaged side (Right:Left:Bilateral)	30:27:3	19:11:0	4:3:2	7:8:0
Microscope during surgery (Yes:No) ^g^	21:32	16:11	1:7	3:9
Enucleation possible during surgery (Yes:No) ^h^	32:19	25:4	3:3	4:10
Perioperative nerve injury (Yes:No) ^i^	12:46	7:21	2:7	2:13
Complications during surgery (Yes:No) ^j^	0:59	0:29	0:9	0:15
Both MRI and PAD performed	39	27	3	9
Radicality in PAD (Yes:No) ^k^	14:17	8:10	0:3	6:4

Basic characteristics of patients surgically treated for a preoperatively suspected benign nerve tumour and with a confirmed diagnosis of Schwannoma, neurofibroma or another tumour in the upper limb. Data presented as n (%), mean (SD), or median [IQR].

^a^ PAD missing in 6 cases.

^b^ Others include the following diagnosis (one of each if not otherwise indicated): glomus tumour (two patients), malignant spindle cell tumour, tendon sheath fibroma, pyogenic granuloma, fibroma, palmar fibromatosis (Dupuytren´s contracture; three patients), angioleiomyoma, benign vessel tumour, lymphangioma, giant cell tumour, vessel anomaly and inconclusive PAD.

^c^ 14 missing cases, of which five were Schwannomas, five neurofibromas, two other diagnoses and two PAD missing.

^d^ Three missing cases, of which one was Schwannoma, one other diagnosis and one PAD missing.

^e^ Two missing cases, both of which PAD missing.

^f^ 13 missing cases, of which two were Schwannomas, four neurofibromas, three other diagnoses and four PAD missing.

^g^ Seven missing cases, of which three were Schwannomas, one neurofibroma and three other diagnoses.

^h^ Nine missing cases, of which one were a Schwannoma, three neurofibromas, one other diagnosis and four PAD missing.

^i^ Two missing cases, both of which were Schwannomas.

^j^ One missing case of Schwannoma.

^k^ 29 missing cases, of which 12 were Schwannomas, six neurofibromas, five other diagnoses and six PAD missing.

### Probabilities, sensitivity, and specificity using MRI for correct diagnosis of Schwannoma

**Fig. 2:**
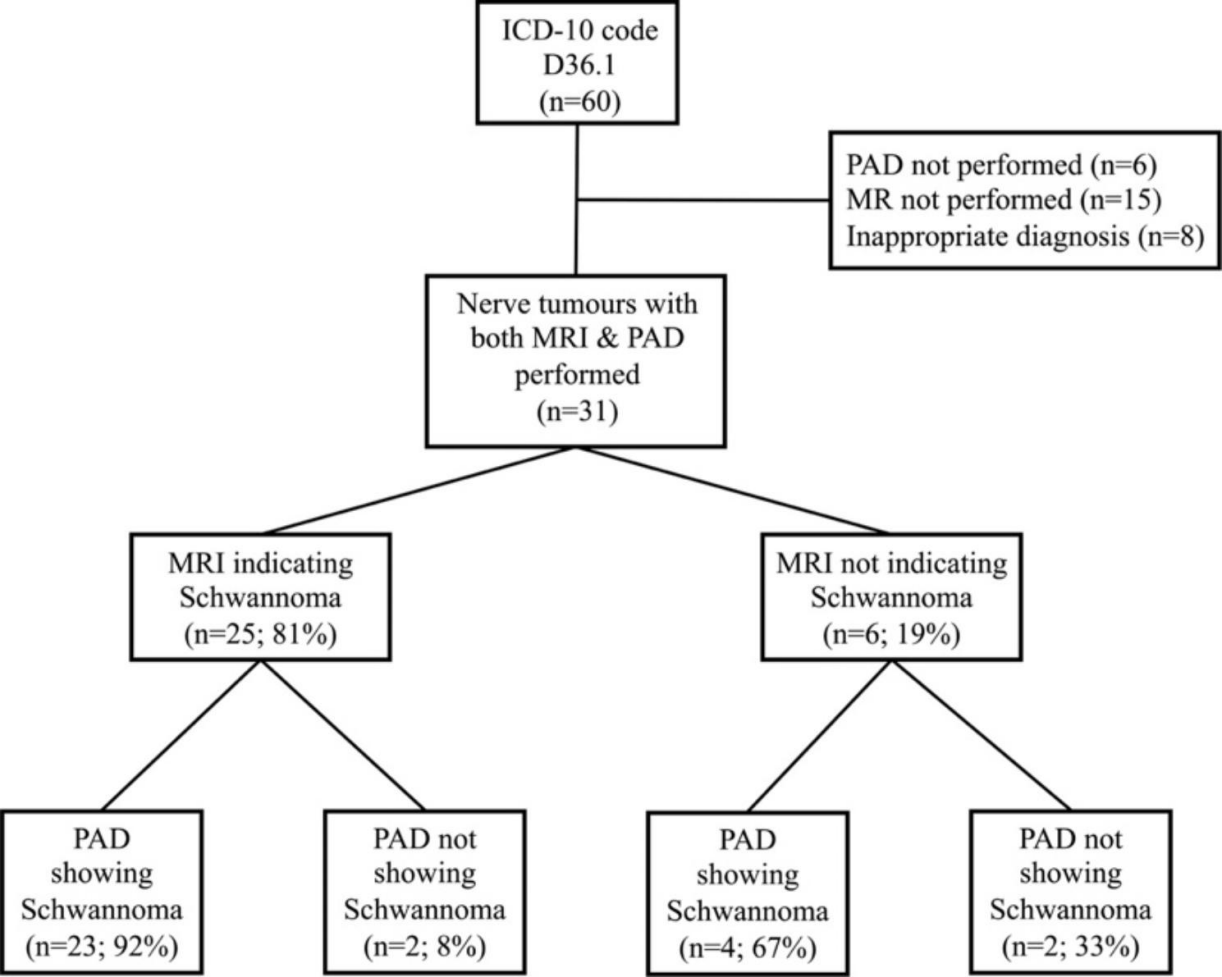
Consistency between preoperative Magnetic Resonance Imaging and pathoanatomical diagnosis of Schwannomas in the upper limb. PAD = Pathoanatomical diagnosis; MRI = Magnetic Resonance Imaging

Altogether, among the patients with a PAD-verified nerve tumour (n = 46/52, 88%, missing n = 6, exclusion of inappropriate diagnosis n = 8), a preoperative MRI was performed in 31/46 (67%) patients. A flowchart of the consistency between MRI and PAD is provided in Fig. 2. The sensitivity of an MRI to correctly identify a Schwannoma in the study population was 85% [23/(23 + 4)] and the specificity was 50% [2/(2 + 2)]. The PPV for MRI to predict a true Schwannoma was 92% [23/(23 + 2)] and the NPV that a given MRI correctly dismissed the diagnosis of Schwannoma was 33% [2/(2 + 4)].

### Location of Schwannoma

The location of the tumour in cases with a PAD-verified Schwannoma ranged from the fingers of the hand up to the brachial plexus (Table 1). The most frequent location of Schwannoma was the forearm (12/30, 40%), followed by the hand (n = 7), elbow level (n = 2), the upper arm (n = 6), shoulder level (n = 2), and a case engaging the brachial plexus (n = 1). The engaged nerve was identified in 28/30 cases (93%), where the median nerve was predominantly affected (n = 15/28, 54%), and the ulnar nerve secondly most affected (n = 10/28, 36%) (Table 1).

### Size of Schwannomas and its relation to symptoms and outcome

**Fig. 3:**
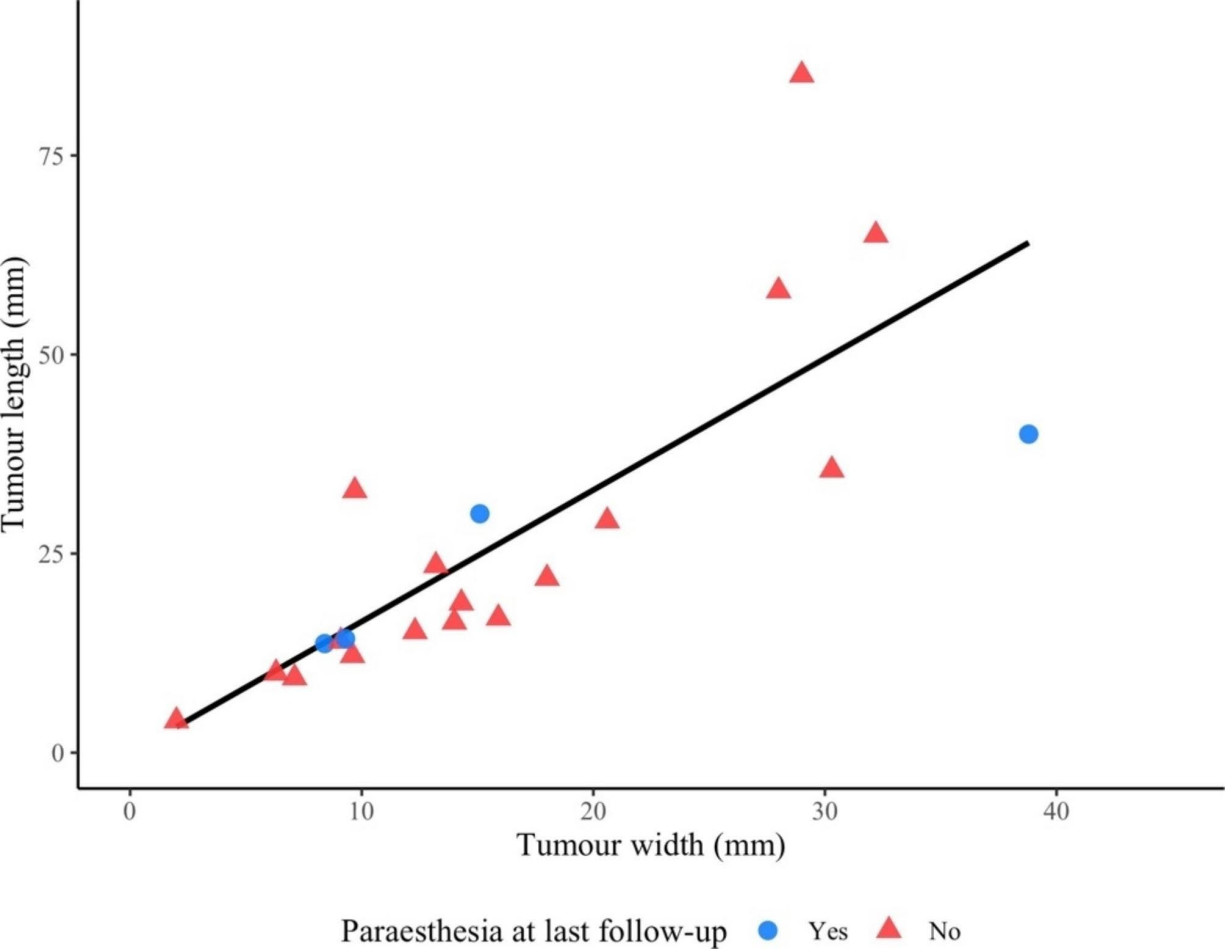
Correlation between width and length in Magnetic Resonance Images of confirmed Schwannomas in the upper limb. Correlation calculated by Spearman´s correlation: r = 0.901, p < 0.001, n = 21

**Fig. 4:**
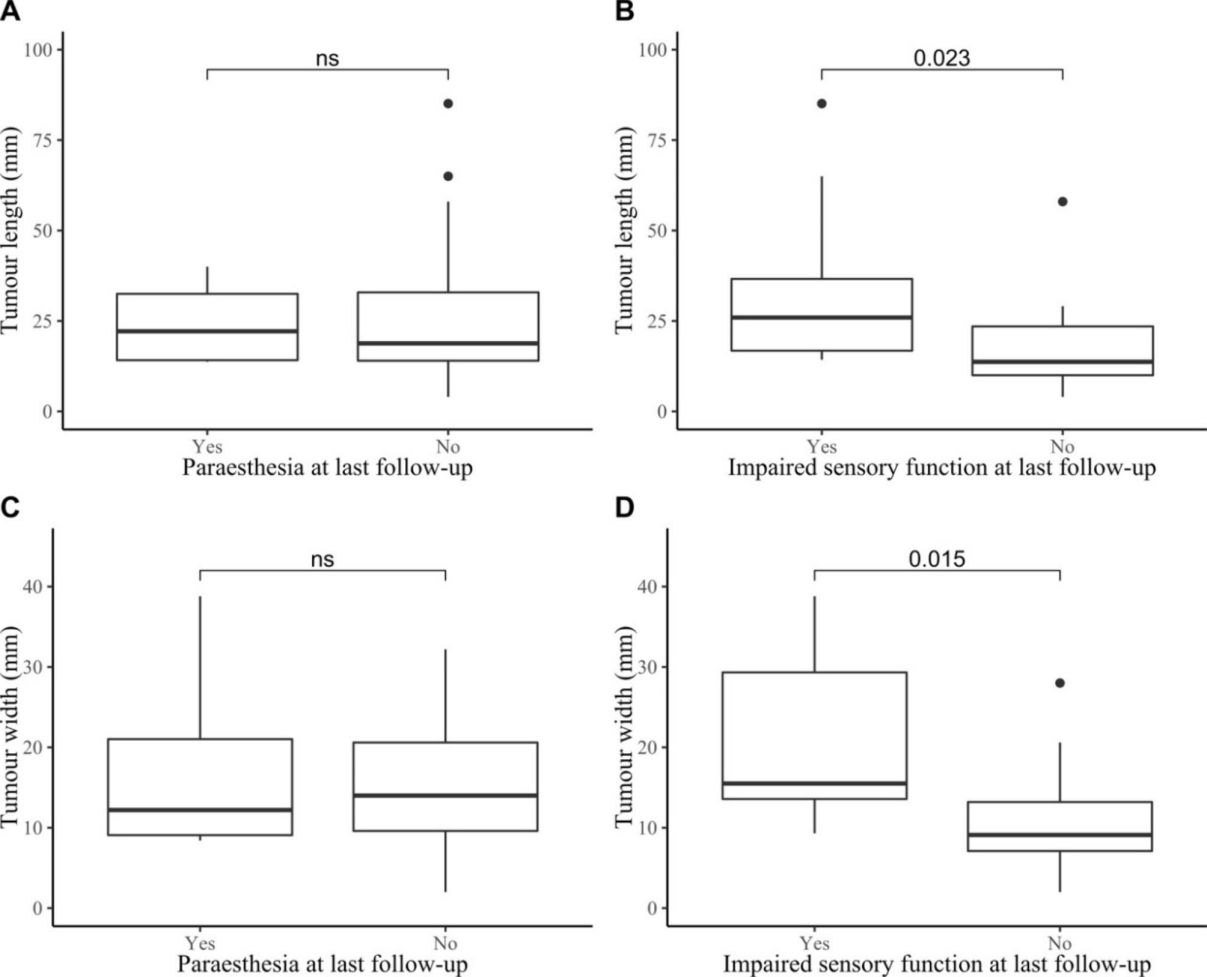
Comparison of tumour size in Schwannomas in the upper limb and association with postoperative paraesthesia and impaired sensory function. Comparison of tumour size in Schwannomas between patients with and without postoperative paraesthesia and impaired sensory function at last follow-up (n = 21). ns = not significant

Regarding tumour size of the Schwannoma measured in the MRI pictures, 25/27 (93%) preoperative MRIs were available for tumour measurements. The median length of the Schwannomas was 19 mm [IQR 14–31 mm] and median width was 14 mm [IQR 9–21 mm]. There was a strong significant correlation (n = 21, r = 0.901, p < 0.001) between the length and width of the Schwannomas (Fig. 3). The length and width of the Schwannomas are presented in Table 2 allocated by symptoms and presence of a palpable tumour. Length and width of the Schwannoma on preoperative MRI were significantly longer among patients with impaired sensory function at last follow-up (n = 21, p = 0.023 and p = 0.015, respectively) (Fig. 4). No size differences were noted between patients with or without the other symptoms at the last follow-up (Table 2).

**Table 2:** Tumour size in relation to pre- and postoperative symptoms and presence of a palpable tumour in patients surgically treated for a PAD verified Schwannoma in the upper limb

	Yes				No				Difference between groups (p-value)	
	n	Length	Width	Ratio	n	Length	Width	Ratio	Length	Width
**Preoperative**	24									
Paraesthesia	21	16 [13–31]	13 [9–21]	1.5 [1.2–2.0]	3	19 [*]	16 [*]	1.2 [*]	0.93	0.50
Impaired sensory function	7	16 [9–33]	14 [7–21]	1.4 [1.2–2.9]	17	19 [14–28]	14 [9–21]	1.3 [1.2–1.7]	0.80	0.62
Impaired motor function	2	21 [*]	15 [*]	1.5 [*]	22	18 [14–31]	14 [10–21]	1.3 [1.2–2.0]	1	0.80
Palpable tumour	24	18 [14–30]	14 [9–21]	1.4 [1.2–1.9]	0					
Pain	15	17 [12–36]	13 [8–28]	1.5 [1.2–2.0]	9	19 [15–28]	14 [11–17]	1.2 [1.2–1.8]	0.68	0.91
**Last follow-up**	21									
Paraesthesia	4	22 [14–38]	12 [9–33]	1.6 [1.2–1.9]	17	19 [13–34]	14 [9–24]	1.4 [1.2–2.0]	0.97	1
Impaired sensory function	12	26 [17–39]	16 [13–30]	1.3 [1.2–2.0]	9	14 [10–26]	9 [7–17]	1.6 [1.4–1.9]	**0.023**	**0.015**
Impaired motor function	9	22 [14–62]	18 [9–31]	1.5 [1.2–2.0]	12	18 [11–30]	14 [8–16]	1.5 [1.2–1.9]	0.35	0.25
Palpable tumour	0				21	19 [14–34]	14 [9–24]	1.5 [1.2–2.0]		
Pain	7	17 [14–40]	16 [8–28]	1.3 [1.1–1.6]	14	20 [14–34]	14 [9–21]	1.6 [1.3–2.0]	1	0.64

Value as median and [IQR] expressed in mm. Ratio is length divided by width. P-values are based on Mann-Whitney U-test. Bold values indicate statistically significant difference

### Symptoms of Schwannoma and outcome of surgery

Pre- and postoperative symptoms and outcome of surgical treatment of the Schwannomas are presented in Table 3. Preoperative symptoms could be assessed in 29 patients with PAD-verified Schwannomas and symptoms at last follow-up after surgical treatment could be assessed in 23 patients. All patients (n = 29/29, 100%) with a Schwannoma had a palpable tumour preoperatively and most patients suffered from symptoms of paraesthesia (n = 24/29, 83%) and pain in the affected area (n = 20/29, 69%). These symptoms were significantly improved at last follow-up. Impaired sensory and motor functions were uncommon preoperatively (n = 8/29, 28% and n = 2/29, 7%), but were present, although not significantly different, at last follow-up (impaired sensory function: n = 14/23, 61%, p = 0.15; impaired motor function: n = 9/23, 39%, p = 0.07) (Table 3). The time since surgery to the last follow-up extended between three and 18 months. Seven patients did not appear at any postoperative consultation (n = 7/30, 23%). Most patients (n = 15/30, 50%) were followed for three months postoperatively, while six patients were followed for six months and one for 12 and 18 months, respectively.

A surgical microscope was used in more than half of the surgeries (n = 16/27, 59%), when approaching the tumour and excision of the Schwannoma, and enucleation was most frequently possible (n = 25/29, 86%). No major perioperative and postoperative complications, such as bleeding, infections, or difficulties in wound healing, were reported. No Complex Regional Pain Syndrome (CRPS) was observed, but some prolonged pain was reported (n = 2/30, 7%). No immediate reoperations were performed, but one patient was operated after 18 months due to a recurrent Schwannoma and one after five years for a new Schwannoma at another localisation. A perioperative nerve injury occurred in 7/28 (25%) cases (missing data in n = 2). Perioperative nerve injuries were more commonly described in the medical records when only using loop magnification and not a surgical microscope (n = 4/10, 40% and n = 2/16, 13%, respectively; p = 0.031). However, there was no statistically detected difference regarding postoperative symptoms between patients surgically treated with and without a surgical microscope (data not shown).

**Table 3:** Pre- and postoperative symptoms (last follow-up) and occurrence of a palpable tumour in patients surgically treated for a PAD-verified Schwannomas in the upper limb

Symptoms	Preoperative (n = 29)		Last follow-up (n = 23)		Difference between groups (n = 22)
	Yes	No	Yes	No	P-value
Paraesthesia	24 (83)	5 (17)	4 (17)	19 (83)	**< 0.001**
Sensory dysfunction	8 (28)	21 (72)	14 (61)	9 (39)	0.15
Motor dysfunction	2 (7)	27 (93)	9 (39)	14 (61)	0.07
Palpable tumour	29 (100)	0 (0)	0 (0)	23 (100)	**< 0.001**
Pain	20 (69)	9 (31)	7 (30)	16 (70)	**0.012**

Data presented as n (%). P-values based on McNemars test and bold indicates statistically significant difference. PAD = Pathoanatomical diagnosis

## Discussion

The present study, consisting of cases with PAD-verified Schwannomas and neurofibromas, and not involving any other nerve tumours or malignant nerve sheath tumours, in the upper limb, shows that the preoperative MRI has a rather high sensitivity (85%), but a low specificity (50%). Paraesthesia and pain improve significantly by surgery, but patients still suffered from relatively high proportions of impaired sensory and motor function postoperatively. Length and width measurements of the tumour on MRI strongly correlated and patients with impaired postoperative sensory function had tumours with greater tumour size.

Our findings indicate a clear compatibility between making the diagnosis from a preoperative MRI based on the available technique at the time of the study and the biopsy report (PAD) for the surgically treated Schwannomas, which is clinically beneficial in treating upper limb Schwannomas. A preoperative MRI shows a high ability to correctly identify and predict Schwannomas in the upper limb when the MRI is performed for a suspected nerve tumour. Similar results have been produced by Adani et al., where the sensitivity of preoperative MRI, correctly identifying Schwannomas, was 91% compared to the percentage in the present study (85%) [22]. Furthermore, recent functional MRI investigations utilising diffusion-weighted imaging and apparent diffusion coefficient are superior for discerning between malignant and benign peripheral nerve tumours compared to conventional MRI [28, 30, 31]. A diagnosis made by a preoperative MRI seems of greater clinical importance when the MRI indicates a Schwannoma with a symptomatic profile suggestive of a benign peripheral nerve tumour [30–32]. One should in this context also consider the value of the anatomical location of the Schwannoma in the peripheral nerve trunk and to the surrounding tissue, with the aid of tractography, which may benefit the surgical planning when a Schwannoma is suspected. However, the study is characterized by lacking presence of other rare nerve tumours, such as lipofibromatous hamartomas, glomus tumours, and perineuriomas, as only Schwannomas and neurofibromas were included in the study [2, 30].

It was possible to determine the tumour size, expressed as length and width of the Schwannoma from the preoperative MRI. A strong correlation was found between tumour length and width, where Schwannomas were axially longer than wide. The size of the Schwannomas had no impact on preoperative paraesthesia, impaired sensory or motor dysfunction, and pain or if the tumour was palpable. However, both the length and width of the Schwannoma influenced the postoperative sensory function, but not paraesthesia, pain, or motor dysfunction, of the affected nerves at the last follow-up visit.

Generally, there were no complications by the surgery, such as infection, bleedings, signs of postoperative fibrosis or severe neurogenic pain. No patients developed CRPS to be compared to previous studies showing 2–5% in surgery for carpal tunnel syndrome and ulnar nerve compression [33, 34].

Enucleation of the Schwannoma was possible in most of the patients. There was a significant difference in perioperative nerve injury between patients having surgery, where the surgeon used a surgical microscope, compared to if surgery was performed with the aid of only loop magnification. However, use of a surgical microscope did not render any further improvements in postoperative symptoms. Furthermore, in accordance with previous studies, the Schwannomas were located almost entirely in the median and ulnar nerves, mainly located in the forearm, which explains particularly the present postoperative sensory dysfunction due to the nerve fibre composition in those nerves [2, 3, 22]. The symptoms paraesthesia, and pain, as well as the experience of a palpable tumour in the nerve trunk, improved significantly by the surgical treatment to the last follow-up visit compared to the preoperative evaluation. However, the preoperatively impaired sensory and motor functions did not significantly change by surgery. Constraining factors were the limited number of cases as per the rarity of the diagnoses and the retrospective design with a rather wide collection time, compromising uniformity between the continuously developing MRI methods used for each patient.

## Conclusions

We conclude that a preoperative MRI is of certain clinical value for accurate preoperative diagnosis and planning of surgery for the nerve tumours Schwannoma and neurofibroma in the upper limb. Recent and future development of the MRI will surely increase its utility and precision in diagnosis of nerve tumours. Overall, patients benefit from surgery of Schwannoma with a low risk for complications, but patients should be informed preoperatively of the risk for a perioperative nerve injury. The tumour size of a Schwannoma is related to the remaining postoperative sensory dysfunction, but patients with greater tumours still benefit from surgery concerning paraesthesia and pain, indicating that surgery should be performed without a long delay.

## Data Availability

Data summaries used to support the findings of this study are included within the article. Complete data accessibility is restricted because of legal and ethical concerns, involving patient privacy and Swedish Ethical Review Authority.

## List of abbreviations

CRPS: Complex Regional Pain Syndrome
IQR: Interquartile Range
MPNST: Malignant peripheral nerve sheath tumour
MRI: Magnetic Resonance Imaging
NPV: Negative predictive value
PAD: Pathoanatomical Diagnosis
PPV: Positive predictive value
SD: Standard deviation
